# Blunted light exposure amplitudes in patients with opioid use disorder

**DOI:** 10.1038/s44323-026-00102-3

**Published:** 2026-07-27

**Authors:** Rebecca C. Cox, Delainey L. Wescott, Alyssa Week, Sophia Haller, Zofia Martinez-Lisowska, Abhignya Kuppa, Melissa P. Knauert, Kathryn A. Roecklein, Sangchoon Jeon, Declan T. Barry, Dustin Scheinost, Nancy S. Redeker, H. Klar Yaggi, Kenneth P. Wright

**Affiliations:** 1 Department of Psychological and Brain Sciences, Washington University in St. Louis, St. Louis, MO USA; 2 Department of Integrative Physiology, University of Colorado Boulder, Boulder, CO USA; 3 Department of Psychiatry, University of Pittsburgh, Pittsburgh, PA USA; 4 Yale School of Medicine, New Haven, CT USA; 5 Department of Psychology, University of Pittsburgh, Pittsburgh, PA USA; 6 Yale University School of Nursing, Orange, CT USA; 7 University of Connecticut Elisabeth DeLuca School of Nursing, Storrs, CT USA; 8 Clinical Epidemiology Research Center VA CT Healthcare Center, West Haven, CT USA

**Keywords:** Diseases, Health care, Medical research, Neuroscience, Physiology

## Abstract

Accumulating evidence implicates sleep and circadian rhythm disruption in substance use disorders, including opioid use disorder (OUD). To understand whether weaker light exposure time cues are observed in patients with OUD, we compared the amplitudes of personal light exposure in patients with OUD (*n* = 73) and healthy controls (*n* = 49). Participants monitored personal light exposure and rest/activity via wrist-worn actigraphy for 1 week. We calculated light and physical activity amplitude for each day with non-orthogonal spectral analysis. Patients with OUD demonstrated significantly lower light exposure amplitudes and significantly higher physical activity amplitudes compared to healthy controls, suggesting lower light exposure amplitudes are not accounted for by sedentary behavior in patients with OUD. Blunted light exposure amplitudes, indicative of a weaker time cue to the circadian clock, characterize patients with OUD and may represent a novel target for improving sleep and circadian health in OUD.

## Introduction

Opioid use disorder (OUD) is a chronic, relapsing condition influenced by a complex array of factors ranging from genetic heritability to mental health to social norms^1^. Over 26.8 million people are estimated to live with OUD worldwide, with the highest prevalence and health burden rates found in the United States (US)^2^. Indeed, in 2019, 49,860 individuals died of an opioid overdose, accounting for 70.6% of drug overdose deaths in the US^3^. OUD is also associated with immense economic costs in the US, with total OUD and fatal opioid overdose costs estimated at $1.02 trillion in 2017^4^. Though medications for opioid use disorder (MOUD), such as methadone, buprenorphine, and naltrexone, are efficacious for treating OUD^5^, MOUD is limited by high drop-out rates, lack of access to care, and stigma^1,6^. Thus, it is critical to identify novel, accessible treatment targets to improve outcomes for individuals with OUD.

One potential target is sleep and circadian rhythms. Prior work has implicated sleep and circadian rhythm disruption in physical and mental health conditions^7^, and sleep and circadian rhythm disruption has been conceptualized as a transdiagnostic risk factor for psychopathology^8^. Likewise, a developing body of work indicates bidirectional relations between sleep and circadian rhythm disruption and substance use^9,10^. Recent perspectives suggest that sleep and circadian rhythm disruption may contribute to the onset of substance use disorders beginning in adolescence^10,11^. Developmental delays in circadian rhythms, coupled with developmental changes in neural systems that regulate reward function^10,11^, confer risk for engaging in substance use behaviors. Further, a recent study found evidence for shared genetic underpinnings of substance use, including OUD, and sleep and circadian disruption^12^.

However, most of the work in sleep, circadian rhythms, and substance use has examined alcohol use and alcohol dependence^9,10^, and considerably less is known about possible sleep and circadian rhythm disruption associated with opioid use and OUD. Given the ongoing opioid crisis, is it crucial to extend the existing literature on sleep and circadian rhythm disruption in substance use disorders broadly to OUD specifically to identify novel treatment targets. A developing body of work has linked sleep and circadian rhythm disruption to OUD, including evidence of short sleep duration^13^, later sleep timing^14^, altered brain transcriptomic and proteomic sleep-related and circadian signaling^15^, and poor sleep quality^16–18^. Subjective sleep disturbance has also been associated with functional outcomes in OUD, including increased pain^17–19^, negative affect^17,18^, and unemployment^16,18^, as well as clinical outcomes including increased craving^20^, return to use, and non-fatal overdose^21^.

Importantly, sleep and circadian rhythms are regulated, in part, by light. Light exposure is the primary time cue for the human circadian clock^22–25^, and the timing and intensity of light exposure influences multiple indicators of circadian timing in humans, including the timing of the melatonin rhythm^26^ and the timing of the sleep-wake cycle^27^. A weaker light circadian time cue (i.e., more time indoors and exposure to electrical light at night) results in later timed circadian clocks and later chronotypes^25^. Recent lighting recommendations to promote circadian and sleep physiology and overall health highlight the need for brighter days and dimmer nights^28^. Amplitude of 24 h light exposure provides a metric of the strength of the time cue provided by light to the circadian clock. Prior findings indicate that lower light amplitude is associated with irregular^29^ and later^30^ sleep timing and later dim light melatonin onset^30^, the primary biomarker of the human circadian clock. Relevant to the current study, individual differences in personal light exposure are modifiable treatment targets.

Limited work has examined the association between personal light exposure and substance use disorders, though accumulating work links unhealthy patterns of light exposure to mental illness broadly^31,32^. This is a notable gap in the literature, as light exposure has been implicated in mood^33^ and reward processes^34^, two mechanisms underlying substance use disorders^11^. Further, significant advances have been made in the study of personal light exposure in recent decades^28^, and the availability of wearable devices to measure ambulatory light exposure in participants natural environments now makes feasible the characterization of this important entrainment cue in complex populations, such as OUD. We therefore compared light exposure amplitudes measured by actigraphy in patients with OUD who were in the first six months of MOUD treatment to healthy controls. We hypothesized that patients with OUD would demonstrate lower daily light exposure amplitudes compared to healthy controls. Given that actigraphy measures light at the wrist and is associated with diurnal physical activity rhythms in other patient samples^35^, it is possible that any differences observed in light exposure amplitudes could be due to differences in physical activity. However, little is known about diurnal physical activity amplitude in patients with OUD. We therefore also examined differences in daily physical activity amplitudes between patients with OUD and healthy controls as an exploratory aim.

## Results

### Descriptive differences between groups

**Fig. 1:**
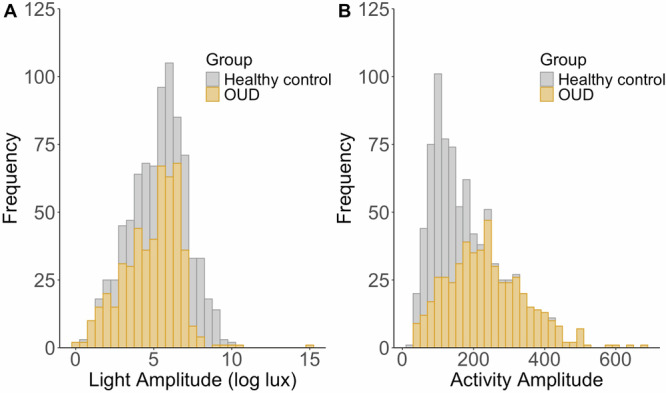
Distributions of light exposure and physical activity amplitudes in patients with opioid use disorder(OUD) and healthy controls (*N* = 122). **A** Distribution of daily light exposure amplitudes in patients with OUD (yellow) and healthycontrols (gray). Bars represent 0.5 log lux bins. **B** Distribution of daily physical activity amplitudes in patients with OUD (yellow) and healthy controls (gray). Bars represent 20 activity count bins.

Descriptive statistics and group comparisons of patients with OUD and healthy controls are shown in Table 1 and distributions are shown in Fig. 1. Compared to healthy controls, patients with OUD were significantly more likely to be male and unemployed and less likely to be Asian (*p*’s < 0.01). Age (*p* = 0.11) and photoperiod did not significantly differ between the two groups (*p* = 0.06). Effect sizes were small to medium (see Table 1).

**Table 1:** Descriptive data by group.

	OUD (*n* = 73)	Healthy controls (*n* = 49)	Test statistic
Age	41.48 (11.49)	37.88 (12.92)	*t*(122) = 1.61, *p* = 0.11, *d* = 0.30
Sex			$\chi$^2^(1,122) = 11.97, *p* < 0.001, φ = 0.31
Female	32 (44%)	37 (76%)	
Male	41 (56%)	12 (24%)	
Race			*p* < 0.01, *V* = 0.34
White	58 (79%)	34 (69%)	
Asian	0 (0%)	7 (14%)	
Black/African American	4 (5%)	5 (10%)	
More than 1 race	11 (15%)	3 (6%)	
Employment status			$\chi$^2^(1,113) = 24.50, *p* < 0.001, φ = 0.47
Employed	20 (27%)	32 (65%)	
Unemployed	51 (70%)	10 (20%)	
Unknown	2 (3%)	7 (14%)	
Photoperiod	12.92 (2.04)	12.25 (1.81)	*t*(120) = 1.89, *p* = 0.06, *d* = 0.35
Latitude	41.8°	40.4°	
Light exposure amplitude	4.88 (1.24)	5.84 (1.46)	
Physical activity amplitude	236.73 (85.44)	116.55 (33.14)	

Percentages that do not total to 100% are due to rounding. Chi square test for employment status did not include unknowns.

### Differences in daily light exposure amplitudes between groups

**Fig. 2:**
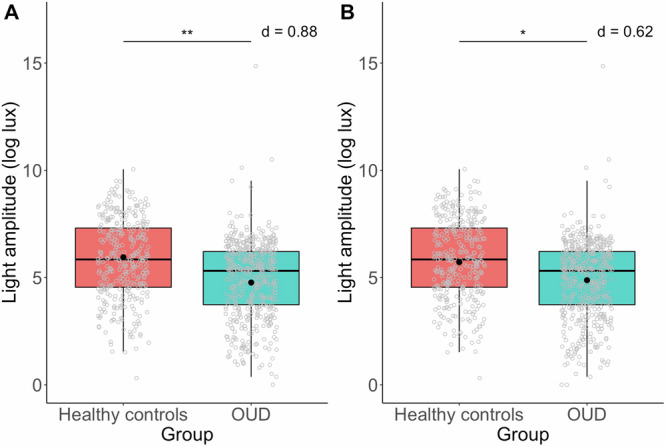
Differences in light exposure amplitudes between patients with opioid use disorder (OUD) and healthy controls. **A** Differences in light exposure amplitudes between patients with opioid use disorder (OUD) and healthy controls unadjusted for demographic covariates. **B** Differences in light exposure amplitudes between patients with opioid use disorder (OUD) and healthy controls adjusted for demographic covariates. Boxes represent 25 and 75 percentiles, and lines represent 1.5*interquartile range. Thick black circles represent estimated marginal means, and thick black lines represent medians. Effect size d = Cohen’s d. **p < 0.001, *p < 0.05.

Results of the models unadjusted for demographic covariates revealed a large, significant effect of group (*p* < 0.001, _p_η^2^ = 0.20), such that healthy controls exhibited 1.25 times higher daily light exposure amplitudes (*M* = 5.95, *SE* = 0.17) compared to patients with OUD (*M* = 4.77, *SE* = 0.14, *d* = 0.88; Fig. 2). There was also a large, significant effect of photoperiod (*p* < 0.001, _p_η^²^ = 0.26), such that longer photoperiods were associated with higher daily light exposure amplitudes, regardless of group. There was not a significant effect of study day (*p* = 0.62, _p_η^²^ < 0.01), suggesting light exposure amplitudes were relatively stable during the sampling period. There was also not a significant effect of day type (*p* = 0.19, _p_η^²^ < 0.01), suggesting light exposure amplitudes did not differ between weekdays and weekends. See Table 2.

**Table 2:** Results of mixed model ANOVAs examining differences in daily light exposure and physical amplitudes between patients with opioid use disorder and healthy controls, controlling for photoperiod, study day, and day type in Model 1 and photoperiod, study day, day type, and demographic covariates in Model 2

	Light exposure amplitudes				Physical activity amplitudes			
	*Df*	*F*	*p*	_p_η^2^	*df*	*F*	*p*	_p_η^2^
Group	1, 117.59	29.96	<0.001	0.20	1, 118.68	84.16	<0.001	0.41
Photoperiod	1, 119.45	41.26	<0.001	0.26	1, 121.36	0.04	0.84	<0.01
Study day	1, 723.17	0.25	0.62	<0.01	1, 723.82	<0.01	0.97	<0.01
Day type	1, 712.98	1.72	0.19	<0.01	1, 718.35	4.44	<0.05	0.01
	Light exposure amplitudes				Physical activity amplitudes			
	*Df*	*F*	*p*	_p_η^2^	*df*	*F*	*p*	_p_η^2^
Group	1, 103.43	9.15	<0.01	0.08	1, 104.86	105.65	<0.001	0.50
Photoperiod	1, 105.11	33.30	<0.001	0.24	1, 106.89	0.63	0.43	0.01
Study day	1, 669.17	0.67	0.41	<0.01	1, 670.96	0.09	0.76	<0.01
Day type	1, 659.07	3.59	0.06	<0.01	1, 664.66	2.72	0.10	<0.01
Sex	1, 104.17	0.10	0.76	<0.01	1, 104.89	2.72	0.10	0.03
Race	3, 104.04	1.68	0.18	0.06	3, 104.90	0.26	0.85	0.01
Employment status	1, 104.68	4.19	<0.05	0.04	1, 105.11	18.44	<0.001	0.15

After adjusting for demographic covariates, the effect of group remained significant (*p* < 0.01, _p_η^2^ = 0.08), such that healthy controls exhibited 1.17 times higher daily light exposure amplitudes (*M* = 5.72, *SE* = 0.23) compared to patients with OUD (*M* = 4.88, *SE* = 0.24, *d* = 0.62) over and above the effects of sex, race, and employment status (Fig. 2). Likewise, the effect of photoperiod also remained significant (*p* < 0.001, _p_η^2^ = 0.24). There were no significant effects of sex (*p* = 0.76, _p_η^²^ < 0.01), race (*p* = 0.18, _p_η^²^ = 0.06), study day (*p* = 0.41, _p_η^²^ < 0.01), or day type (*p* = 0.06, _p_η^²^ < 0.01). However, after adjusting for demographic covariates, there was a significant effect of employment status (*p* < 0.05, _p_η^2^ = 0.04), such that daily light exposure amplitudes were higher in those who were employed (*M* = 5.56, *SE* = 0.23) compared to those who were unemployed (*M* = 5.04, *SE* = 0.23, *d* = 0.38). See Table 2.

### Differences in daily physical activity amplitudes between groups

**Fig. 3:**
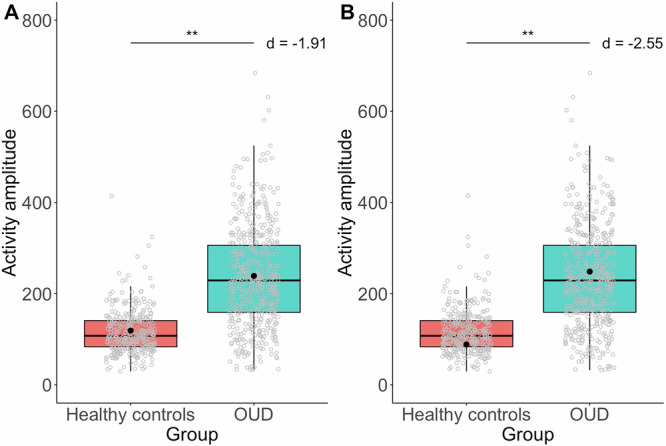
Differences in physical activity amplitudes between patients with opioid use disorder (OUD) and healthy controls. **A** Differences in physical activity amplitudes between patients with opioid use disorder (OUD) and healthy controls unadjusted for demographic covariates. **B** Differences in physical activity amplitudes between patients with opioid use disorder (OUD) and healthy controls adjusted for demographic covariates. Boxes represent 25 and 75 percentiles, and lines represent 1.5*interquartile range. Thick black circles represent estimated marginal means, and thick black lines represent medians. Effect size d = Cohen’s d. **p < 0.001.

Results of the models unadjusted for demographic covariates revealed a large, significant effect of group (*p* < 0.001, _p_η^2^ = 0.41), such that patients with OUD exhibited 2.01 times higher daily physical activity amplitudes (*M* = 239, *SE* = 8.26) compared to healthy controls (*M* = 119, *SE* = 10.10, *d* = -1.91) (Fig. 3). There was not a significant effect of photoperiod (*p* = 0.84, _p_η^²^ < 0.01) or study day (*p* = 0.97, _p_η^²^ < 0.01), suggesting physical activity exposure amplitudes were relatively stable across seasons and during the sampling period, respectively. However, there was a small, significant effect of day type (*p* < 0.05, _p_η^2^ = 0.01), such that daily activity amplitudes were higher on weekends (*M* = 184, *SE* = 7.29) compared to weekdays (*M* = 174, *SE* = 6.58, *d* = 0.16). See Table 2.

After adjusting for demographic covariates, the effect of group remained significant (*p* < 0.001, _p_η^2^ = 0.50) such that patients with OUD exhibited 2.80 times higher daily physical activity amplitudes (*M* = 248.40, *SE* = 13.50) compared to healthy controls (*M* = 88.60, *SE* = 13.20, *d* = −2.55) over and above the effects of sex, race, and employment status. There were no significant effects of sex (*p* = 0.10, _p_η^²^ = 0.03), race (*p* = 0.85, _p_η^²^ = 0.01), photoperiod (*p* = 0.43, _p_η^²^ = 0.01), study day (*p* = 0.76, _p_η^²^ < 0.01), or day type (*p* = 0.10, _p_η^²^ < 0.01). There was also a large, significant effect of employment status (*p* < 0.001, _p_η^2^ = 0.15), such that employed participants exhibited 1.44 times higher daily physical activity amplitudes (*M* = 199, *SE* = 13.10) than unemployed participants (*M* = 138, *SE* = 12.80, *d* = 0.95). See Table 2.

## Discussion

The current study provides a novel assessment of habitual light exposure patterns in patients with OUD compared to a matched sample of healthy controls. Consistent with our hypothesis, we found that patients with OUD had blunted light exposure amplitudes compared to healthy controls, which likely reflects a weaker entraining signal to the circadian clock. Targeted interventions to improve healthy lighting environments in patients with OUD may be a novel and accessible treatment to improve overall functioning and well-being.

Interestingly, a recent study comparing light exposure between patients with OUD and healthy controls found no differences between the groups in light timing or mean amount of daytime (defined as 5 am to 9 pm) and nighttime (defined as 9 pm to 5 am) light exposure^36^. Our ability to detect a difference in light exposure between patients with OUD and healthy controls in the present study may be due to calculating light amplitude by harmonic regression, which accounts for the full 24 h day without creating arbitrary daytime and nighttime bins that may not reflect the individuals behavior. Alternatively, the discrepant findings between studies may reflect differences in duration of treatment in the OUD groups. The study by Zhang & colleagues included individuals with OUD who had received MOUD for a mean of 4 years, whereas our study included individuals with OUD who recently began MOUD. Thus, the absence of differences in personal light exposure observed in the former study may reflect improvements in circadian health during MOUD, whereas those in our study may not have been on MOUD long enough to achieve potential improvements in circadian health. Future longitudinal research tracking indicators of circadian health over time during MOUD may help clarify these discrepant findings.

Blunted light exposure amplitude weakens the entraining signal to the circadian clock, likely leading to disrupted circadian rhythms. In animal models, greater daytime light exposure associates with higher firing rates in the central circadian clock, signaling a more robust circadian system^37^. The greater separation between daytime and nighttime light intensities (e.g., “brighter days and darker nights”^28^) is important for less variable and earlier timed circadian rhythms^22^. Blunted light amplitude weakens the distinction between day and night leading to a weaker environmental time cue and often more variable and later timed circadian rhythms. Animal models also suggest that irregularity of light patterns increases the sensitivity of the circadian system to environmental perturbations^38^. In humans, ample daytime light reduces the sensitivity of the circadian system to the delaying effects of evening light^39,40^. Taken together, blunted patterns of light exposure in patients with OUD may lead to a weakened and more sensitive circadian system which in turn may contribute to the sleep disruptions evident in OUD, including short sleep duration^13^, later sleep timing^14^, altered brain transcriptomic and proteomic sleep-related and circadian signaling^41^, and poor sleep quality^16–18^. Notably, MOUD, including methadone and buprenorphine, produce opioid miosis, or pupil constriction due to opioid agonists^42,43^. Thus, patients with OUD receiving MOUD are likely receiving less light input to the circadian system than healthy individuals due to a combination of lower exposure to ambient light levels and diminished retinal irradiance as a result of smaller pupil diameter.

We also found higher physical activity amplitudes in patients with OUD compared to healthy controls. Though few studies have examined diurnal physical activity patterns in patients with OUD, our finding is consistent with a recent study that likewise found higher physical activity amplitude in patients with OUD compared to healthy controls, despite also finding higher sleep-wake irregularity in OUD compared to healthy controls^36^. Higher physical activity amplitudes in patients with OUD may reflect factors encompassed by social determinants of health. For example, employed patients with OUD may be more likely to hold physically demanding jobs compared to healthy controls, unemployed patients with OUD may be vulnerable to being unhoused, and patients with OUD may have less access to personal transportation regardless of employment status^44,45^. Alternatively, the higher physical activity amplitudes observed in patients with OUD may be an artefact of receiving outpatient MOUD. Indeed, regular visits to the clinic to receive MOUD may increase and stabilize activity; thus, our findings may overestimate activity amplitude relative to patients with OUD who are not receiving MOUD. Although we might expect factors that increase physical activity amplitudes (e.g., being unhoused, using public transit) to also increase personal light exposure, it should be noted that the amplitude incorporates both the highest and lowest points of an oscillation; thus, lower light amplitudes combined with higher physical activity amplitudes may reflect a pattern of well-consolidated physical activity (e.g., high physical activity during the day, low physical activity at night) with poorly consolidated light exposure (e.g., moderate light exposure during the day and at night). Importantly, the higher activity amplitudes observed in patients with OUD suggests that the blunted light exposure amplitudes are not better accounted for by being sedentary compared to healthy controls.

Personal light exposure is a modifiable intervention target. Targeted lighting interventions, such as increasing daytime light^46,47^ and reducing nighttime light after sunset, could increase light exposure amplitude and potentially enhance circadian robustness. Notably, prior work suggests morning bright light therapy reduces pain and functional impairment in individuals with fibromyalgia^48^, suggesting the utility of light therapy for other pain-relevant conditions, such as OUD. Intervening on sleep-wake regularity may also be a modifiable, behavioral intervention to increase light amplitude. Healthy light exposure patterns are associated with regular sleep-wake times^49^, whereas blunted daytime light and increased evening light are associated with sleep-wake irregularities^29^. Stabilizing sleep-wake behaviors, such as maintaining consistent wake times and a morning walk outside or exposure to morning sunlight through a window and dimming indoor lighting after sunset before bedtime, could potentially increase light amplitude. Implementing low-stigma, accessible sleep and lighting interventions in patients with OUD may be a promising strategy to improve sleep and circadian health with possible downstream effects on secondary symptoms (e.g., pain and fatigue).

Strengths of this study include a large sample size and objective real-world measurement. However, the findings of this study must be considered within the context of the study limitations. The sample was predominately white, which limits the ability to generalize these findings to other race and ethnicity groups. Similarly, the OUD group, who were engaged in treatment and stable enough to provide 7 days of actigraphy data, may not reflect all adults with OUD. Data were collected from individuals with OUD during the COVID-19 pandemic, which may have impacted behavior. A recent study found substantially lower personal light exposure in female versus male adults^50^; thus, our findings may underestimate group differences in light exposure amplitude given that female participants were overrepresented in the healthy control group. Further, a known limitation of actigraphy-assessed light exposure is that light measured at the wrist likely differs from light input to the eye. Relatedly, though we made efforts to exclude apparent invalid observations, it is possible that light exposure amplitudes were underestimated due to participant nonadherence to actigraphy instructions (e.g., covering the device with a sleeve). Importantly, there are other approaches to modeling amplitude which could also be appropriate for constructing daily light exposure amplitudes^51,52^. It should also be noted that our approach, which applied harmonic regression to calculate amplitude for individual days, does not account for irregularity between days or the timing of light exposure. Given that circadian sensitivity to light is largely driven by melanopic photoreception^25,53^, future studies are needed with newer wearable devices that provide estimates of melanopic EDI lux, a metric that quantifies light exposure in terms of impact on melanopsin^28^. Future studies are also needed to describe population norms for light exposure amplitude, other factors that impact light exposure amplitude, and to determine how light exposure amplitude levels are associated with health and risk of other diseases. Despite these limitations, our findings suggest that blunted light exposure amplitudes, indicative of a weaker time cue to the circadian clock, characterize patients with OUD and may represent a novel target for improving sleep and circadian health in OUD.

## Methods

### Participants

This analysis examined participants that particated in one of two separate studies. The studies reported here were approved by the Yale University Institutional Review Board (2000026681) and the University of Pittsburgh Institutional Review Board (STUDY20040069). Informed consent was obtained from all participants, and the study was conducted in accordance with standard ethical protocols.

#### OUD

Seventy-nine adults with OUD recently stabilized on outpatient MOUD (42% female (*n* = 33), 75% white (*n* = 59), *M*_age_ = 41.56, *SD* = 11.67) were recruited from two methadone treatment clinics operated by the Addiction Prevention and Treatment (APT) Foundation in Connecticut: New Haven and North Haven (latitude 41°18’N). Participants completed baseline from 2021 to 2024 for enrollment in the Collaboration Linking Opioid Use Disorder and Sleep (“CLOUDS”) study, an NIH HEAL-funded longitudinal study to evaluate sleep and circadian mechanisms that contribute to nonmedical opioid use and MOUD retention (U01HL150596). Included participants were 18 years of age or older, English-speaking, met DSM-5 critieria for OUD, and were receiving MOUD for up to 6 months. The study was open to APT patients receiving methadone or buprenorphine; however, all CLOUDS participants were receiving methadone (baseline methadone dose *M* = 80.03 mg, *SD* = 25.51 mg). All patients at the APT Foundation completed a diagnostic evaluation at intake by an APT clinician. OUD diagnosis was confirmed in CLOUDS participants by a research assistant who administered the NIDA Clinical Trials Network (CTN) DSM-5 Substance Use Symptom Checklist. Participants were excluded for acute psychosis, acute homicide or suicide risk, if they required acute hospitalization for a medical or psychiatric condition, required prescription opioids, had a pending legal action or upcoming relocation that would impede participation, were pregnant, breastfeeding, or unwilling to use birth control (female participants of child-bearing age), currently receiving positive airway pressure therapy, doing shift work, or had contraindications for fMRI scanning. Participants provided written informed consent. The study was approved by the Institutional Review Board at Yale School of Medicine and the APT Foundation.

#### Healthy controls

Forty-nine healthy control adults (76% female (*n* = 37), 69% white (*n* = 34), *M*_age_ = 37.88, *SD* = 12.92) were recruited from the Pittsburgh, PA community from the Pitt+Me participant registry through the University of Pittsburgh (latitude 40°26′N) from 2016 to 2020. Participants were enrolled in the Melanopsin Photosensitivity and Psychopathology (MAPP) study, an NIH-funded longitudinal study to evaluate seasonal variation in light sensitivity in psychopathology (R01MH103313). Participants were excluded for psychotic disorders, bipolar disorders, self-reported sleep disordered breathing, narcolepsy, substance use disorders, self-reported shiftwork >12 times in the past year, transmeridian travel within 1 month, and retinal pathologies including color blindness measured with the Ishihara Test. All study procedures were approved by The University of Pittsburgh Institutional Review Board, and the research was conducted in accordance with the Helsinki Declaration as revised in 1989.

### Procedure

This study is a secondary analysis of an ongoing study of sleep and circadian rhythms in OUD. The parent study does not include a healthy control group; therefore, a healthy control group was selected to be similar to the OUD group in terms of age, actigraphy device, and latitude, with the latter factor being relevant for matching the groups on photoperiod and environmental light intensity. An appropriate healthy control comparison group was identified from the MAPP archival dataset at the University of Pittsburgh.

Participants completed one week of ambulatory monitoring via an actigraph (Actiwatch Spectrum, Respironics) worn on the non-dominant wrist to record illuminance and rest/activity. Wrist-worn light meters are valid measures of ambient light^54^. Though not a laboratory-grade photometer, the measurement error is consistent within devices^55^. The Actiwatch measures illuminance from color sensitive (red, green, blue) photodiodes in photopic lux with a reported measurement range of 5 to 100,000 lux (10% accuracy at 3000 lux). The Actiwatch measures physical activity from a MEMS-type accelerometer in counts per minute and detects acceleration between 0.5 and 2 G. We used the “white” light output (representing a near-linear combination of the output of the red, green, and blue photodiodes)^56^ and activity counts output (i.e., counts per minute). Illuminance and activity were sampled in 60 s epochs in patients with OUD and in 30 s epochs in healthy controls. An amplitude value for light exposure and physical activity was calculated for each study day using non-orthogonal spectral analysis, a harmonic regression model that fits a combination of sine and cosine waves and a serial correlated error term^57^. We applied 3 harmonics to the data, allowing variance over 6 h, which is consistent with harmonic regression approaches for modelling melatonin^24,25^. Data from patients with OUD was resampled at a rate of 1 per every 6 epochs, and data from healthy controls was resampled at a rate of 1 per every 12 epochs to account for differences in sampling rate. The calculated amplitude is a composite of the fitted harmonics and represents the distance from mesor to peak, per circadian field standard nomenclature. Thus, a doubling of the amplitude values reported here would represent the peak to trough amplitude. Missing values and days with more than 4 hours of missing data were removed prior to amplitude calculation^58^. Days with known missing data (first and last day of data collection where device was not worn for a full 24-hr period) and invalid epochs and instances of non-wear resulting in missing data (identified by Phillips Actiware software) were also removed prior to amplitude calculation. Many metrics can be calculated to quantify personal light exposure^25,58,59^. We chose amplitude as a comprehensive metric that reflects the strength of the entraining signal to the circadian clock.

The photoperiod for each study day in New Haven/North Haven, CT and Pittsburgh, PA was calculated as the duration of time between sunrise and sunset, which were retrieved from the National Oceanic and Atmospheric Administration’s Solar Calculator (https://gml.noaa.gov/grad/solcalc/). Data were collected year-round in the OUD group and in winter (Dec 21-Mar 21) and summer (Jun 21-Sept 21) in the healthy control group.

### Statistical analysis

We conducted statistical analysis in R version 4.4.1. Six participants with OUD with less than 7 days of data were excluded, resulting in a final sample *n* = 73 participants with OUD. Days with light exposure amplitude values < 1 (suggesting invalid data, e.g., actigraph covered with a sleeve) were removed from analysis, resulting in the removal of 6 single day instances from the OUD group. Tests of model assumptions revealed heteroscedasticity in the model testing group differences in light exposure amplitude. We therefore log transformed light exposure amplitudes.

We examined differences in age and photoperiod between patients with OUD and healthy controls with an independent samples *t*-test, differences in sex and employment status with chi square tests, and differences in race with a Fisher’s exact test. We examined differences in daily light exposure and physical activity amplitudes between patients with OUD and healthy controls with linear mixed model ANOVAs using the *lmer* package with a random effect of subject and fixed effects of study day (i.e., day 1, day 2…day 7), day type (weekend vs weekday), photoperiod, and group (unadjusted models). We then tested models adjusted for demographic factors that significantly differed between groups, including sex, race, and employment status (adjusted models). We calculated partial η^2^ to evaluate effect sizes for model main effects using standard thresholds (0.01 = small, 0.06 = medium, 0.14 = large) and Cohen’s *d* to evaluate effect sizes for marginal mean differences between groups using standard thresholds (0.2 = small, 0.5 = medium, ≥ 0.80 = large).

Seven participants (*n* = 4 OUD, *n* = 3 healthy controls) participated during the biannual time change (*n* = 2 during fall transition to Standard Time; *n* = 5 during spring transition to Daylight Saving Time). Excluding these participants did not change the pattern of results; therefore, these participants were retained in analyses.

## Data Availability

Datasets generated and/or analysed during the current study are not publicly available due to ongoing data analysis on primary study aims but are available from the corresponding author on reasonable request.

## References

[1] Strang, J. et al. Opioid use disorder. *Nat. Rev. Dis. Prim.* **6**, 3 (2020). 31919349 10.1038/s41572-019-0137-5

[2] GBD 2016 Disease and Injury Incidence and Prevalence Collaborators Global, regional, and national incidence, prevalence, and years lived with disability for 328 diseases and injuries for 195 countries, 1990-2016: A systematic analysis for the Global Burden of Disease Study 2016. *Lancet* **390**, 1211–1259 (2017). 28919117 10.1016/S0140-6736(17)32154-2 PMC5605509

[3] Mattson, C. L. et al. Trends and geographic patterns in drug and synthetic opioid overdose deaths-United States, 2013 – 2019. *MMWR Morb. Mortal. Wkly Rep.* **70**, 202–207 (2021). 33571180 10.15585/mmwr.mm7006a4 PMC7877587

[4] Florence, C., Luo, F. & Rice, K. The economic burden of opioid use disorder and fatal opioid overdose in the United States, 2017. *Drug Alcohol Depend.* **218**, 108350 (2021). 33121867 10.1016/j.drugalcdep.2020.108350 PMC8091480

[5] Volkow, N. D., Frieden, T. R., Hyde, P. S. & Cha, S. S. Medication-assisted therapies: tackling the opioid-overdose epidemic. *N. Engl. J. Med.* **370**, 2061–2063 (2014). 24758595 10.1056/NEJMp1402780

[6] Volkow, N. D., Jones, E. B., Einstein, E. B. & Wargo, E. M. Prevention and treatment of opioid misuse and addiction: A review. *JAMA Psychiatry* **76**, 208–216 (2019). 30516809 10.1001/jamapsychiatry.2018.3126

[7] Foster, R. G. Sleep, circadian rhythms and health. *Interface Focus* **10**, (2020). 10.1098/rsfs.2019.0098 PMC7202392 32382406

[8] Harvey, A. G., Murray, G., Chandler, R. A. & Soehner, A. Sleep disturbance as transdiagnostic: Consideration of neurobiological mechanisms. *Clin. Psychol. Rev.* **31**, 225–235 (2011). 20471738 10.1016/j.cpr.2010.04.003 PMC2954256

[9] Hasler, B. P., Smith, L. J., Cousins, J. C. & Bootzin, R. R. Circadian rhythms, sleep, and substance abuse. *Sleep. Med. Rev.* **16**, 67–81 (2012). 21620743 10.1016/j.smrv.2011.03.004 PMC3177010

[10] Roehrs, T., Sibai, M. & Roth, T. Sleep and alertness disturbance and substance use disorders: a bi-directional relation. *Pharmacol. Biochem. Behav.* **203**, 173153 (2021). 33582097 10.1016/j.pbb.2021.173153 PMC7996967

[11] Logan, R. W. et al. Impact of sleep and circadian rhythms on addiction vulnerability in adolescents. *Biol. Psychiatry* **83**, 987–996 (2017). 29373120 10.1016/j.biopsych.2017.11.035 PMC5972052

[12] Hatoum, A. S. et al. Characterisation of the genetic relationship between the domains of sleep and circadian-related behaviours with substance use phenotypes. *Addiction Biol.* **27**, 313184 (2022). 10.1111/adb.13184 PMC10038127 35754104

[13] Mukherjee, D. et al. Reregulation of cortisol levels and sleep in patients with prescription opioid use disorder during long-term residential treatment. *Drug Alcohol Depend.* **227**, 108931 (2021). 34392049 10.1016/j.drugalcdep.2021.108931

[14] Bertz, J. W. et al. Sleep reductions associated with illicit opioid use and clinic hour changes during opioid agonist treatment for opioid dependence: Measurement by electronic diary and actigraphy. *J. Subst. Abus. Treat.* **106**, 43–57 (2019). 10.1016/j.jsat.2019.08.011 PMC6756187 31540611

[15] Puig, S. et al. Circadian rhythm disruptions associated with opioid use disorder in synaptic proteomes of human dorsolateral prefrontal cortex and nucleus accumbens. *Mol. Psychiatry* **28**, 4777–4792 (2023). 37674018 10.1038/s41380-023-02241-6 PMC10914630

[16] Baldassarri, S. R. et al. Correlates of sleep quality and excessive daytime sleepiness in people with opioid use disorder receiving methadone treatment. *Sleep. Breath.* **24**, 1729–1737 (2020). 32556918 10.1007/s11325-020-02123-z PMC7680294

[17] Dunn, K. E., Finan, P. H., Tompkins, D. A. & Strain, E. C. Frequency and correlates of sleep disturbance in methadone and buprenorphine-maintained patients. *Addictive Behav.* **76**, 8–14 (2018). 10.1016/j.addbeh.2017.07.016 PMC5614840 28735039

[18] Stein, M. D. et al. Sleep disturbances among methadone maintained patients. *J. Subst. Abus. Treat.* **26**, 175–180 (2004). 10.1016/S0740-5472(03)00191-0 15063910

[19] Ponce Martinez, C. et al. Associations among sleep disturbance, pain catastrophizing, and pain intensity for methadone-maintained patients with opioid use disorder and chronic pain. *Clin. J. Pain.* **36**, 641–647 (2021). 10.1097/AJP.0000000000000848 PMC7725378 32482968

[20] Bichon, J. A., Bailey, A. J., Votaw, V. R. & McHugh, R. K. Sleep disruption, stress, and craving during inpatient treatment for opioid use disorder. *Addictive Behavi.* **170**, (2025). 10.1016/j.addbeh.2025.108427 PMC13535798 40614370

[21] Hochheimer, M. et al. Insomnia symptoms are associated with return to use and non-fatal overdose following opioid use disorder treatment. *Sleep* **48**, (2025). 10.1093/sleep/zsae284 39657100

[22] Duffy, J. F. & Wright, K. P. Entrainment of the human circadian system by light. *J. Biol. Rhythms* **20**, 326–338 (2005). 16077152 10.1177/0748730405277983

[23] Golombek, D. A. & Rosenstein, R. E. Physiology of circadian entrainment. *Physiol. Rev.* **90**, 1063–1102 (2010). 20664079 10.1152/physrev.00009.2009

[24] Wright, K. P., Hughes, R. J., Kronauer, R. E., Dijk, D.-J. & Czeisler, C. A. Intrinsic near-24-h pacemaker period determines limits of circadian entrainment to a weak synchronizer in humans. *Proc. Natl. Acad. Sci. USA.* **98**, 14027–14032 (2001). 11717461 10.1073/pnas.201530198 PMC61161

[25] Wright, K. P. et al. Entrainment of the human circadian clock to the natural light-dark cycle. *Curr. Biol.* **23**, 1554–1558 (2013). 23910656 10.1016/j.cub.2013.06.039 PMC4020279

[26] Khalsa, S. B. S., Jewett, M. E., Cajochen, C. & Czeisler, C. A. A phase response curve to single bright light pulses in human subjects. *J. Physiol.* **549**, 945–952 (2003). 12717008 10.1113/jphysiol.2003.040477 PMC2342968

[27] Komada, Y., Tanaka, H., Yamamoto, Y., Shirakawa, S. & Yamazaki, K. Effects of bright light pre-exposure on sleep onset process. *Psychiatry Clin. Neurosci.* **54**, 365–366 (2000). 11186116 10.1046/j.1440-1819.2000.00717.x

[28] Brown, T. M. et al. Recommendations for daytime, evening, and nighttime indoor light exposure to best support physiology, sleep, and wakefulness in healthy adults. *PLoS Biol.* **20**, e3001571 (2022). 35298459 10.1371/journal.pbio.3001571 PMC8929548

[29] Phillips, A. J. K. et al. Irregular sleep/wake patterns are associated with poorer academic performance and delayed circadian and sleep/wake timing. *Sci. Rep.* **7**, 1–13 (2017). 28607474 10.1038/s41598-017-03171-4 PMC5468315

[30] Van der Maren, S. et al. Daily profiles of light exposure and evening use of light-emitting devices in young adults complaining of a delayed sleep schedule. *J. Biol. Rhythms* **33**, 192–202 (2018). 29463186 10.1177/0748730418757007

[31] Burns, A. C. et al. Day and night light exposure are associated with psychiatric disorders: an objective light study in >85,000 people. *Nat. Ment. Health* **1**, 853–862 (2023).

[32] Paksarian, D. et al. Association of outdoor artificial light at night with mental disorders and sleep patterns among US adolescents. *JAMA Psychiatry* **77**, 1266–1275 (2020). 32639562 10.1001/jamapsychiatry.2020.1935 PMC7344797

[33] Fernandez, D. C. et al. Light Affects Mood and Learning through Distinct Retina-Brain Pathways. *Cell* **175**, 71–84.e18 (2018). 30173913 10.1016/j.cell.2018.08.004 PMC6190605

[34] Itzhacki, J., Lindert, B. H. W., Kringelbach, M. L. & Mendoza, J. Environmental light and time of day modulate subjective liking and wanting. *Emotion* **19**, 10–20 (2019). 29504798 10.1037/emo0000402

[35] Esaki, Y. et al. Habitual light exposure and circadian activity rhythms in bipolar disorder: A cross-sectional analysis of the APPLE cohort. *J. Affect. Disord.* **323**, 762–769 (2023). 36538951 10.1016/j.jad.2022.12.034

[36] Zhang, R. et al. Rest-activity rhythms, their modulators, and brain-clinical correlates in opioid use disorder. *JAMA Netw. Open* e2457976 (2025) 10.1001/jamanetworkopen.2024.57976. 10.1001/jamanetworkopen.2024.57976 PMC11795329 39903462

[37] Bano-Otalora, B. et al. Bright daytime light enhances circadian amplitude in a diurnal mammal. *Proc. Natl. Acad. Sci. USA.* **22**, e2100094118 (2021). 10.1073/pnas.2100094118 PMC8179182 34031246

[38] Leise, T. L. et al. Recurring circadian disruption alters circadian clock sensitivity to resetting. *Eur. J. Neurosci.* **51**, 2343–2354 (2020). 30269396 10.1111/ejn.14179 PMC6441381

[39] Swaminathan, K., Klerman, E. B. & Phillips, A. J. K. Are Individual Differences in Sleep and Circadian Timing Amplified by Use of Artificial Light Sources? *J. Biol. Rhythms* **32**, 165–176 (2017). 28367676 10.1177/0748730417699310 PMC5593073

[40] Chang, A. M., Scheer, F. A. J. L. & Czeisler, C. A. The human circadian system adapts to prior photic history. *J. Physiol.* **589**, 1095–1102 (2011). 21224217 10.1113/jphysiol.2010.201194 PMC3060589

[41] Xue, X. et al. Molecular rhythm alterations in prefrontal cortex and nucleus accumbens associated with opioid use disorder. *Transl. Psychiatry* **12**, (2022). 10.1038/s41398-022-01894-1 PMC8960783 35347109

[42] Walsh, S. L., Preston, K. L., Stitzer, M. L., Cone, E. J. & Bigelow, G. Clinical pharmacology of buprenorphine: Ceiling effects at high doses. **5**, 569–580 (1994). 10.1038/clpt.1994.71 8181201

[43] Weinhold, L. L. & Bigelow, G. E. *Opioid Miosis: Effects of Lighting Intensity and Monocular and Binocular Exposure*. *Drug and Alcohol Dependence* vol. 31 (1993). 10.1016/0376-8716(93)90070-7 8436062

[44] Do, V. M., Simpson, S., Fisch, K. M. & Gabriel, R. A. Associations Between Social Determinants of Health and Opioid-Use Disorder Among Chronic Pain Patients From a Multi-Institutional Dataset. *Anesth. Analg*. (2024) 10.1213/ANE.0000000000007247. 10.1213/ANE.0000000000007247 PMC12183315 39715077

[45] Gazzola, M. G. et al. A national study of homelessness, social determinants of health, and treatment engagement among outpatient medication for opioid use disorder-seeking individuals in the United States. *Subst. Abus.* **44**, 62–72 (2023). 37226909 10.1177/08897077231167291

[46] Walch, O., Tavella, F., Zeitzer, J. M. & Lok, R. Beyond phase shifting: targeting circadian amplitude for light interventions in humans. *Sleep* **48**, (2025). 10.1093/sleep/zsae247 PMC11725520 39435852

[47] Lok, R. et al. Timing of outdoor light exposure is associated with sleep-wake consolidation in community-dwelling older men. *Front. Sleep* **2**, (2023). 10.3389/frsle.2023.1268379 PMC12713814 41426466

[48] Burgess, H. J. et al. A 4-week morning light treatment with stable sleep timing for individuals with fibromyalgia: a randomized controlled trial. *Pain Med.*10.1093/pm/pnad007. (2023). 10.1093/pm/pnad007 PMC10321765 36715638

[49] Hand, A. J. et al. Measuring Light Regularity: Sleep Regularity is Associated with Regularity of Light Exposure in Adolescents. *Sleep*10.1093/sleep/zsad001/6982548. (2023). 10.1093/sleep/zsad001 PMC10424172 36625482

[50] Wallace, D. A. Light exposure differs by sex in the US, with females receiving less bright light. *npj Biological Timing and Sleep* **1**, (2024). 10.1038/s44323-024-00016-y PMC12395419 40895801

[51] Stone, J. E., Postnova, S., Sletten, T. L., Rajaratnam, S. M. W. & Phillips, A. J. K. Computational approaches for individual circadian phase prediction in field settings. *Curr. Opin. Syst. Biol*. **22**, 39–51 (2020).

[52] Windred, D. P. et al. Brighter nights and darker days predict higher mortality risk: A prospective analysis of personal light exposure in >88,000 individuals. *Proc. Natl. Acad. Sci*. *USA*. **121**, (2024). 10.1073/pnas.2405924121 PMC11513964 39405349

[53] Brown, T. M. Melanopic illuminance defines the magnitude of human circadian light responses under a wide range of conditions. *J. Pineal Res*. **69**, (2020). 10.1111/jpi.12655 32248548

[54] Jardim, A. C. N. et al. Validating the use of wrist-level light monitoring for in-hospital circadian studies. *Chronobiol. Int.* **28**, 834–840 (2011). 21936617 10.3109/07420528.2011.611603

[55] Markvart, J., Hansen, ÅM. & Christoffersen, J. Comparison and correction of the light sensor output from 48 wearable light exposure devices by using a side-by-side field calibration method C. *LEUKOS* **11**, 155–171 (2015).

[56] Price, L. L. A., Khazova, M. & O’Hagan, J. B. Performance assessment of commercial circadian personal exposure devices. *Lighting Res. Technol.* **44**, 17–26 (2012).

[57] Czeisler, C. A. et al. Stability, precision, and near – 24-hour period of the human circadian pacemaker. *Science* **2177**, 2177–2182 (1999). 10.1126/science.284.5423.2177 10381883

[58] Reid, K. J. et al. Timing and intensity of light correlate with body weight in adults. *PLoS One* **9**, e92251 (2014). 24694994 10.1371/journal.pone.0092251 PMC3973603

[59] Wescott, D. L. et al. Circadian photoentrainment varies by season and depressed state: associations between light sensitivity and sleep and circadian timing. *Sleep* **47**, (2024). 10.1093/sleep/zsae066 PMC11168757 38530635

